# Plasmid DNA contaminant in molecular reagents

**DOI:** 10.1038/s41598-019-38733-1

**Published:** 2019-02-07

**Authors:** N. Wally, M. Schneider, J. Thannesberger, M. T. Kastner, T. Bakonyi, S. Indik, T. Rattei, J. Bedarf, F. Hildebrand, J. Law, J. Jovel, C. Steininger

**Affiliations:** 1 Division of Infectious Diseases, Department of Medicine 1, Medical University of Vienna, Vienna, Austria; 2 University of Veterinary Medicine, Department of Virology, Vienna, Austria; 3 CUBE-Division of Computational Systems Biology, Department of Microbiology and Ecosystem Science, University of Vienna, Vienna, Austria; 4 German Centre for neurodegenerative disease research (DZNE), Department of Neurology, University of Bonn, Bonn, Germany; 5 European Molecular Biology Laboratory, EMBL, Heidelberg, Germany; 6 Department of Medicine, University of Alberta, Edmonton, Alberta Canada

## Abstract

Background noise in metagenomic studies is often of high importance and its removal requires extensive post-analytic, bioinformatics filtering. This is relevant as significant signals may be lost due to a low signal-to-noise ratio. The presence of plasmid residues, that are frequently present in reagents as contaminants, has not been investigated so far, but may pose a substantial bias. Here we show that plasmid sequences from different sources are omnipresent in molecular biology reagents. Using a metagenomic approach, we identified the presence of the (*pol*) of equine infectious anemia virus in human samples and traced it back to the expression plasmid used for generation of a commercial reverse transcriptase. We found fragments of multiple other expression plasmids in human samples as well as commercial polymerase preparations. Plasmid contamination sources included production chain of molecular biology reagents as well as contamination of reagents from environment or human handling of samples and reagents. Retrospective analyses of published metagenomic studies revealed an inaccurate signal-to-noise differentiation. Hence, the plasmid sequences that seem to be omnipresent in molecular biology reagents may misguide conclusions derived from genomic/metagenomics datasets and thus also clinical interpretations. Critical appraisal of metagenomic data sets for the possibility of plasmid background noise is required to identify reliable and significant signals.

## Introduction

Metagenomics dramatically changed our view on the composition of microbial communities in a diversity of ecosystems, including particularly the gut associated microbiome. The large-scale, indiscriminate sequencing used in metagenomics allows to comprehensively map all microbial components within well-defined ecosystems. However, an important and often ignored pitfall of this sequencing approach is the indiscriminate sequencing of all sequences present in a given sample. Genomic sequences that may have been inadvertently introduced into samples during processing will be sequenced at a similar efficacy as target sequences and this background noise may mask signals obtained from target sequences. This is a common contamination problem, as exemplified in studies that used Whole Genome Amplification (WGA), where as few as 30% of all reads originated from target DNA^1^.

Genomic background noise may originate from very different sources and may be introduced at multiple points during sample preparation. For example, bacteriophage ΦX174 genomic DNA is added to samples as positive control and despite standard filtering of these sequences it may still be found in published metagenomics samples^2^. More commonly, however, genomic background noise may be introduced inadvertently into the test system. For example, cross-species contamination from bacterial and mammalian DNA has been reported frequently from metagenomic studies^3–6^. Background nucleic acids are commonly introduced inadvertently by human handling of samples^7,8^ via air^9,10^, commercial enzymes^11–17^, DNA extraction kits^18–20^, Ultrapure-water Systems (UPW)^21–23^ or paper points^24^. Even plain buffer solutions used in metagenomics may be source of foreign DNA^25^. The ensuing high background noise reduce the generalizability of findings and differentiation from meaningful signals^26–31^. Extensive and stringent post-analytical bioinformatic filtering of data sets is, therefore, required to ensure a clean look at the biological system. Nevertheless, if filtering parameters are too lenient, this would pose the risk of eliminating biologically meaningful signals from data sets.

An important but widely ignored source of foreign genomic sequences are enzyme preparations used for NGS. Enzymes such as polymerases are generated recombinantly in prokaryotic hosts with the usage of an inducible expression vectors. Residual DNA introduced by the protein expression, particularly bacterial DNA, has been reported since the early 90’s^32–37^. The most widely used enzyme, *Taq* polymerase, is estimated to contain between 10^2^ to 10^5^ genome equivalents of bacterial DNA per unit of enzyme^11,38,39^. In addition to bacteria-derived residues, murine^13,40,41^ and human retroviruses^42^ as well as bacterial-phage such as DNA sequences^12^ were identified in further enyzme preparations.

The relevance of residual expression vectors for NGS, however, has not been elucidated so far. Plasmids are naturally occurring circular shaped pieces of extrachromosomal DNA, which can replicate independently from the host DNA and can be transferred into samples via multiple routes^43,44^. Complete expression vector sequences have not been identified in commercially available enzyme preparations although fragments interfered occasionally with sequencing studies^14,15,17,41,42,45^. For example, Tenover *et al*., had to use a native *Taq* polymerase to avoid false-positive test results when evaluating samples for the presence of antibiotic resistance genes such as Bla_Tem-1_ because most expression vectors used for generation of the enzyme also had this resistance gene^46^. The erroneous identification of antibiotic resistance genes potentially has far-reaching consequences such as misguided patient treatment^47^.

In a recent metagenomic virome study, we frequently found signatures of natural plasmids in human samples^48^. More remarkably, we also found sequences of a horse retroviral pathogen in these human samples. The *pol* gene of the Equine Infectious Anemia Virus (EIAV) was found in all urine- and pharyngeal lavage samples collected from healthy human volunteers. EIAV is a species-specific lentivirus that infects *Equidae* and causes an immunodeficiency syndrome similar to that of the Human Immunodeficiency Virus (HIV-1). The immunological, virological and medical implications for the common presence of an equine retrovirus in human samples are far-reaching^49^. Still, viruses in general, are very specific for their hosts and cross-species infections are rare events.

The aim of the present study is, therefore, to evaluate the source and the biological relevance of this finding. Surprisingly, we uncovered a common theme in NGS workflows – introduction of foreign plasmid DNA from very different and multiple sources into samples tested with NGS methods. Our findings may have far-reaching biological consequences for wide areas of life sciences.

## Results

### Equine Infectious anemia virus *pol* sequences are derived from extrinsic plasmids

**Figure 1:**
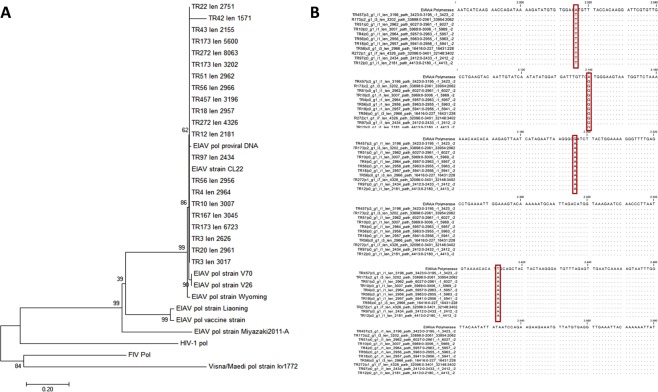
EIAV *pol* Analysis. (**A**) Neighborhood-Joining Tree with Bootstrap resampling of EIAV contigs with following standard strains: EIAV Pol Wyoming (gb|AF016316.1), EIAV Pol Liaoning (gb|AF327877.1), EIAV Pol Vaccine Strain (gb|AF327878.1), EIAV Pol V70 Strain (gi 9929860), EIAV Pol V26 Strain (gi 9929867), EIAV Pol (NC_001450.1), EIAV Pol Clone 22 (M87581.1), EIAV pol Miyazaki2011-A (GenBank: JX003263.1), Human Immunodeficiency Virus-1 pol (HIV-1; NC_001802.1), Feline Immunodeficiency Virus pol (FIV; NC_001482.1) and Maedi/Visna pol strain kv1772 (NC_001452.1). (**B**) Alignment of all preliminary found EIAV pol sequences together with the standard strain EIAV Wyoming (EIAVuk). Although shorter, the sequence shows a high homology to the EIAVuk strain. Furthermore, the sequences itself show no alteration. Differing nucleic acids are boxed in red.

In a previous study, we detected contigs containing the polymerase (*pol*) gene of the retrovirus Equine infectious anemia virus (EIAV) in all evaluated human samples from healthy volunteers (n = 4)^48^. EIAV is a retrovirus infecting *Equidae* but not reportedly humans and also has not been reported as a zoonotic disease of humans so far^49^. A phylogenetic analysis of the sequences found in relation to those of other *lentiviridae* such as Human Immunodeficiency Virus-1 *pol* (HIV-1; NC_001802.1), Feline Immunodeficiency Virus *pol* (FIV; NC_001482.1) and Maedi/Visna *pol* strain kv1772 (NC_001452.1) showed a high similarity of the sequences detected with the *pol* gene of the EIAV clone CL 22 strain (ID: M87581.1; Fig. 1). Further alignment of sequences showed no genetic variation among the *pol* sequence we found, which is highly unusual for retroviruses with high mutation rates. Only when compared to the standard strain EIAV Wyoming, a small number of nucleotide differences had been identified.

**Figure 2:**
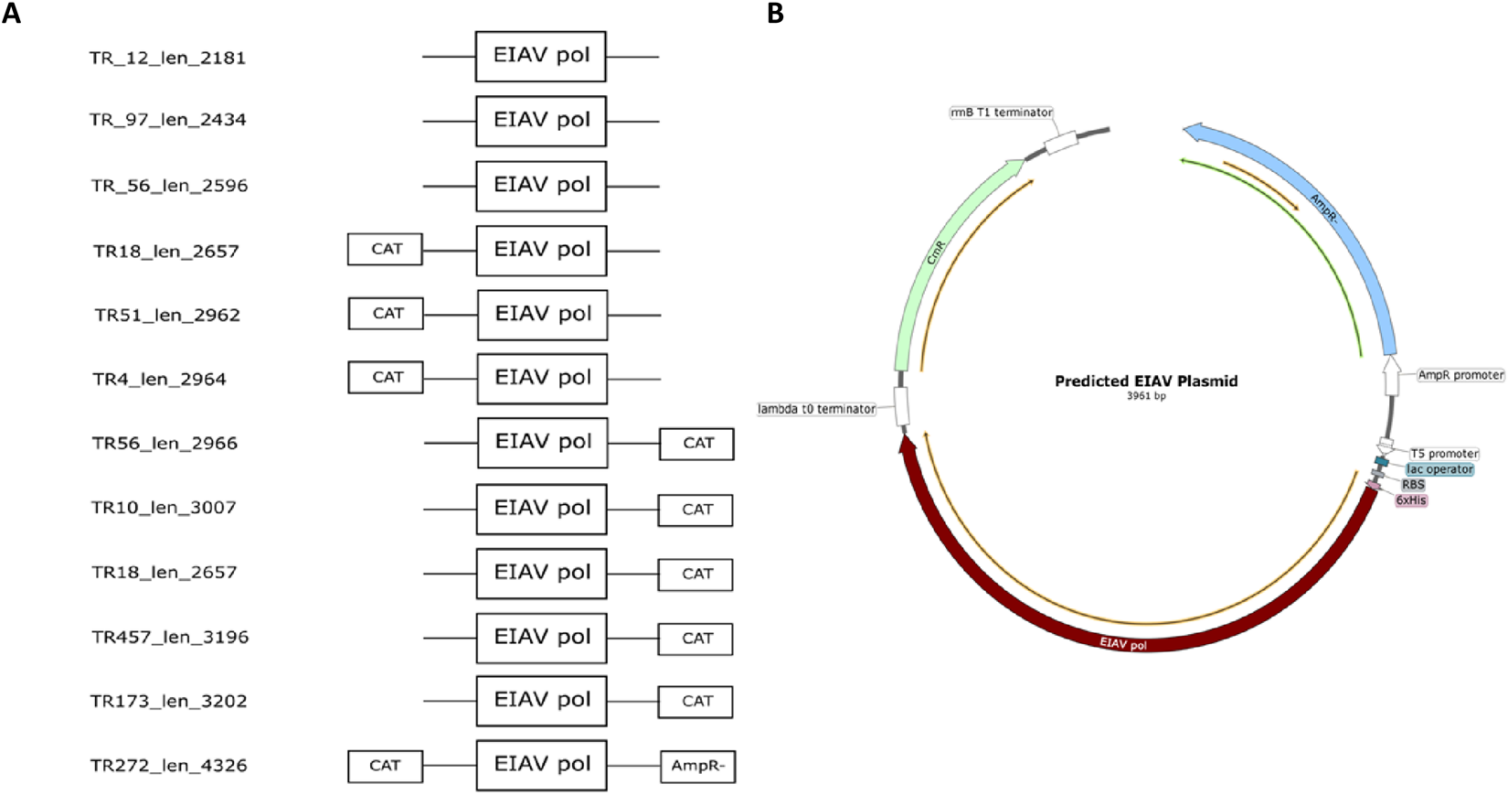
Analysis of EIAV plasmid (**A**) Blast search revealed for sequences above 2.5 kb the presence of a CAT (Chloramphenicolacetyltransferase). For the longest sequence UN_TR272_len_4326 a second bacterial resistance (AmpR-) conferring a resistance to ß-Lactam antibiotics such as Ampicillin. (**B**) Plasmid map of the predicted Omniscript RT Kit expression plasmid which was identified as the source of the EIAV *pol*. Qiagen confirmed that such a plasmid is used for their Omniscript product. The EIAV pol sequence is in-frame with a histidin-tag, flanked by a BamHI and a HindIII restriction site and followed by a lambda t0 terminator. Further downstream a inactive CmR resistance followed by a rrnB T1 Terminator. Further upstream a AmpR promoter together with a ß-lactamase can be found. In front of the Insert is a Ribosomal Binding Site (RBS) with a T5 promoter to ensure strong transcription. The system is induced by a lac operator. The backbone of the plasmid seems to be pDS56/RBSII and therefore the origin of replication may be pBR332. The whole plasmid with the name p6EIAV-RT was created by Dr. Stuart J LeGrice in 1991.

All fragments found, corresponded only to a part of the *pol* gene of EIAV reference strains (1.667 kb). Furthermore, the *pol* sequences identified were flanked by a CmR sequence (Chloramphenicol acetyltransferase; ID: EDS05563.1), and in the case of the longest contig available by an additional Bla_Tem-1_ resistance-encoding sequence (ID: WP_000027050.1, Fig. 2A). Further assembly of EIAV *pol* flanking sequences revealed additional genes indicative for the presence of an expression vector including a Histidine-Tag, a Ribosomal Binding Site (RBS), a lac operator, a T5 promoter and a lambda t0 as well as a rrnB T1 terminator (Fig. 2B).

**Figure 3:**
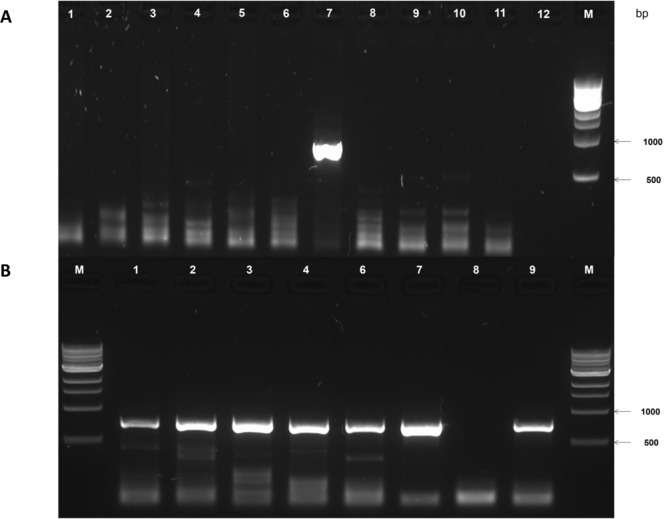
Sample testing of environmental equipment for EIAV. Enriched DNA and RNA tested without (**A**) and with Reverse-Transcription with the Omniscript RT Kit (**B**). When reverse transcribing the samples with Omniscript RT Kit (Qiagen, Hildesheim, Germany), all samples turned positive including the non-template control of the reverse transcription. The negative control of the amplification (Lane B7) was negative and the positive control with the EIAV plasmid was positive (B8). (**A**) Testing of laboratory environment and two patient samples for EIAV. 1: Ultrafiltrated DNA of Pharyngeal lavage Healthy Volunteer C, 2: Ultrafiltrated DNA of Urine Healthy Volunteer J, 3: Ultrafiltrated DNA of Pharyngeal lavage Volunteer J, 4: Adenovirus DNA, 5: Influenza strain A/Puerto Rico/8/1934 H1N1 DNA, 6: EIAV39 cDNA, 7: EIAV Packaging Plasmid containing *gag* and *pol* region (positive control), 8: Mili-Q Water, 9: Distilled Ultrafiltrated Mili-Q Water, 10: Gibco Dulbecco’s Modified Eagle Medium, 11: Gibco Trypsin-EDTA, 12. Gibco Pencillin-Streptomycin Testing the Distilled Ultrafiltrated Mili-Q Water counted also as negative control. (**B**) Testing of samples after Reverse Transcription with Omniscript RT-Kit: 1: Ultrafiltrated DNA of Pharyngeal lavage Healthy Volunteer C, 2: Ultrafiltrated DNA of Urine Healthy Volunteer J, 3: Ultrafiltrated DNA of Pharyngeal lavage Volunteer J, 4: Adenovirus DNA, 5: Influenza strain A/Puerto Rico/8/1934 H1N1 DNA, 6: Non-Template control of Omniscript RT-Kit (1 µl of enclosed Nuclease-free Water as Template), 7: Non-template control of PCR, 8: EIAV Packaging Plasmid containing *gag* and *pol* region (positive control). A Marker on the left side was excluded as the lane was overloaded. Additionally, another experiment below the upper experiment (**A**) was excluded. Raw Data is available in the Supplementary File.

To validate the presence of a vector and to identify the source of contamination, we tested all laboratory consumables and clinical samples used previously by Thannesberger *et al*., with the use of a PCR assay that is specific for the EIAV *pol* sequences found. Surprisingly, all of these samples were negative for EIAV *pol* sequences (Fig. 3A). To exclude the presence of an RNA template of the EIAV *pol* sequences, samples had been tested again after reverse transcription with Omniscript RT Kit (Qiagen, Hildesheim, Germany). After that, all samples that were reverse transcribed had been tested positive for EIAV *pol* sequences, including also the non-template control of the reaction mix (Fig. 3B). Therefore, we suspected that the RT kit used (Omniscript RT Kit) is the EIAV *pol* source. To validate this hypothesis, we treated all of these samples with a different reverse-transcriptase (iScript cDNA Synthesis Kit, Biorad, California, USA) and repeated the same experiment. These experiments yielded uniformly negative test results (data not shown), which further indicates that the Omniscript RT Kit was the source of the EIAV *pol* sequences.

In order to quantify the overall genomic background noise present during the virome testing procedure, a qPCR was designed that is specific for the CmR resistance found frequently in the EIAV contigs. Three different time steps, reflecting the enzymatically treatment incorporated in the standard workflow of the VIPEP method, had been tested and designated T0, T1 and T2. Time step T0 contained the reverse transcription mix (Omniscript RT Kit) without performing reverse transcription, T1 after the reverse transcription, and T2 was after a multiple displacement amplification (MDA) of 1 µl T1 with REPLI-g Mini Kit (Qiagen, Hildesheim, Germany). The plasmid copy number increased from 39,249 per µl at T0 to 383,045 copies at T1 and 245,444,045 copies at T2.

### Characterization of omnipresent natural and artificial plasmid residues in NGSs reagents

**Figure 4:**
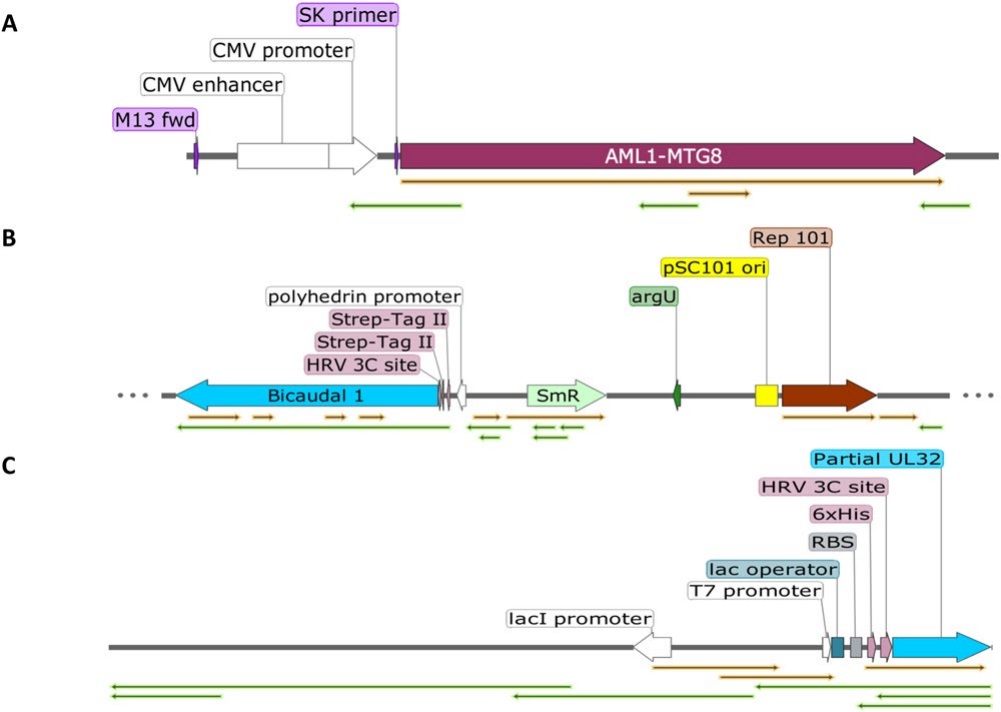
Predicted sequences of other expression vectors found (**A**) Predicted AML-1 MT8 Plasmid containing a truncated Cytomegalovirus promoter (CMVd2) with a M13 forward primer Site and a SK-Primer site in front of a 2,2 kb long open reading frame coding for a protein which showed a 99% query cover with homo sapiens mRNA for AML1-MTG8 fusion protein (GenBank: D13979.1). A similar published expression vector (Miyoshi *et al*., 1993) would explain the SK primer but not the CMVd2 promoter. (**B**) Predicted Bicaudal 1 Plasmid contains a rep101 origin of replication (Gene Bank: sp|P22308.1|REPY_ECOLX). The original origin of replication of this vector is a high-copy number f1 ori, however no f1 ori’s were found. Furthermore, another gene was exchanged. Instead of an aminoglycoside 3′-phosphotransferase (Gene Bank: P00552.1) exhibiting kanamycin/neomycin resistance a Streptomycin 3″-adenylyltransferase (Gene Bank: sp|P0AG05.1|S3AD_ECOLX) was found. (**C**) Partial UL-32 Plasmid Together with the BicD1 vector, a UL-32 expression vector was co expressed. Very few, incomplete fragments were found. It seems to be mixed up with parts of the BicD1 vector (e.g. HRV 3C Site). The blast search for the insert, gave a hit for the UL-32 gene of the HSV-5 (Cytomegalovirus) strain AD169, complete genome (gb|FJ527563.1|).

**Figure 5:**
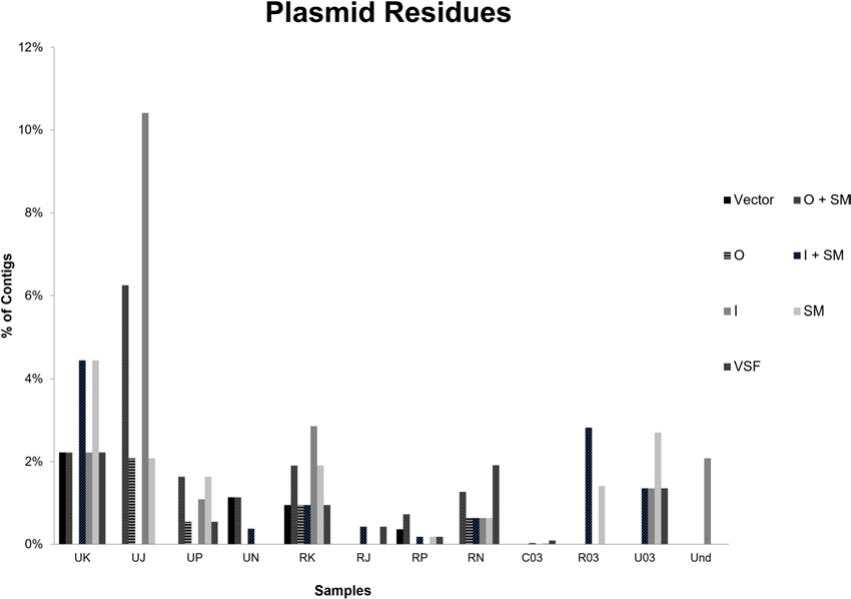
Statistical evaluation and definition of plasmid contamination. Statistical evaluation and definition of plasmid contamination. Plasmid sequences not fulfilling the criteria mentioned are designated “artificial fragmented plasmids” The complete definition for an artificial fragmented plasmid is as following: “May contain several artificial sequences similar to a complete vector but is missing one criteria which can be: ori (O), selection marker (SM), promoter region with insert (I) regardless length and is not naturally occurring”. Due to the nature of fragmented plasmids, they may have either one or two features and are further characterized by them (e.g. ori with selection marker = O + SM). Sequences containing neither an ori, selection marker or insert but contained any other plasmid feature (e.g. histidine-tags) were termed very short fragments (VSF). A total of 80 plasmid sequences were found, representing 1.61% of all contigs. The total plasmid contamination varied from 0.16% in our control group up to 20.83% in a patient sample UJ. UK: Urine Volunteer K, UJ: Urine Volunteer J; UP: Urine Volunteer P; UN: Urine Volunteer N, RK: Nasopharyngeal Lavage Volunteer K, RJ: Nasopharyngeal Lavage Volunteer J, RP: Nasopharyngeal Lavage Volunteer P, RN: Nasopharyngeal Lavage Volunteer N, C03: Our Non-Template Control, R03: Technical Replicate Nasopharyngeal Lavage, U03: Technical Replicate Urine, Und: Undetermined Sequences.

After that, all contigs available from the previous study had been re-evaluated *in silico* for the presence of plasmid sequences such as selection markers and origin of replication to evaluate the possible presence of additional artificial expression vectors. We found multiple other sequences exhibiting characteristics of expression vectors (Fig. 4). Of 4956 contigs from twelve samples, 1.61% (n = 80) contained plasmid sequences. These sequences were found in such diverse samples such as human urine (n = 4), pharyngeal lavages (n = 4), technical replicate groups (n = 2) and a non-template control (n = 1). The relative abundance of plasmid background ranged from 0.16% in the Non-Template Control (NTC) up to 20.83% in one patient sample. Interestingly, the urine samples had a higher plasmid background with a mean of 11.67% (Max: 20.83%; Min: 2.65%; SD: 8.97%) compared to the pharyngeal lavage samples with a mean of 4.67% (Max: 10.47%; Min: 2.65%; SD: 4.42%). The urine technical replicates had higher plasmid residues compared to the pharyngeal lavage technical replicates (6.757% vs. 4.225%) (Fig. 5).

#### Characterization of plasmid residues

Of the 80 contigs with plasmid signatures, 41% (n = 33) had an origin of replication, 63% (n = 51) a selection marker and 52% (n = 42) an insert. Apart from the EIAV coding expression vector, three other artificial expression vectors could be identified by their inserts. Of these inserts, 19% included a chimera of a human-mouse chimera Bicaudal 1 gene (n = 8), 11% the UL-32 gene of the Cytomegalovirus (n = 5) and 5% the leukemia fusion protein AML1-MTG8 (n = 2). All contigs with a specific insert had been aligned and the consensus sequence displayed in SnapGene Viewer gave a predicted plasmid map (Fig. 5). The plasmids coding for Bicaudal 1 chimera and UL-32 genes were identical to those used for other studies in our laboratory and had, therefore, been identified as laboratory contaminants. BLAST of the 2268 bp long fragment of “Und_TR29_len2635”, found in the Und sample (Undetermined contigs), showed a 99% query coverage with homo sapiens mRNA for AML1-MTG8 fusion protein (GenBank: D13979.1). The source of this plasmid remains unknown.

#### Natural plasmids residues are derived from a variety of sources

Besides the presence of artificial plasmids, natural occurring plasmids from different species were found in all twelve samples (n = 12). The most frequent plasmid was from *Micrococcus spp*. (92%) followed by *Serratia spp*. (50%), *Burkholderia spp*. (42%), *Ralstonia spp*. (25%), *Acinetobacter spp*. (25%), *Mucilaginibacter spp*. (17%), *Streptococcus spp*. (17%), *Enterobacter spp*. (8%) and *Cupriavidus spp*. (8%; Table 1). The plasmid sequences we found from *Serratia maracesens* pUO901 (ID: NG_047232.1) and *Enterobacter cloacae* pEC005 (ID: NG_050201.1) coded only for antibiotic resistances. The first one was identified as a aminoglycoside-(3)-N-acetyltransferase (AAC(3)s), whereas the latter coded for a Class A extended-spectrum beta lactamase TEM-157 (Table 1). These plasmids are likely from natural sources.

**Table 1:** Distribution of natural and artificial Plasmids in samples used by Thannesberger *et al*.^48^.

Plasmids	Sample ID											
	UK	UJ	UP	UN	RK	RJ	RP	RN	C03	U03	R03	Und
**Natural Plasmids**												
*Burkholderia cepacia* PPC1	+		+		+			+	+			
*Micrococcus Luteus* pMLU1	+	+	+	+	+	+	+	+	+	+	+	
*Serratia marcescens* pUO901	+	+	+	+	+		+					
*Mucilaginibacter* sp. PAMC		+			+							
*Campylobacter concisus* pCCON16		+										
*Ralstonia pickettii* 12D pRp12D02			+	+					+			
*Enterobacter cloacae* pEC005 Class A ESBL TEM-157				+								
*Cupriavidus metallidurans* CH34 megaplasmid									+			
*Streptococcus pneumoniae* pSpnP1										+		+
*Acinetobacter baumannii* str. AYE plasmid p1ABAYE									+		+	+
**Artificial Plasmids**												
EIAV Vector Residues	+	+	+	+	+	+	+	+	+		+	
Bicaudal 1 Vector Residues	+	+		+	+		+	+				
AML1-MTG8 Vector Residues										+		
UL-32 Vector Residues		+	+		+							+

Burkholderia cepacia PPC1 (ID: NC_010099.1), Micrococcus luteus pMLU1 (ID: NC_003526.1), Serratia marcescens pUO901 aac(3)-I gene for AAC(3)-I family aminoglycoside 3-N-acetyltransferase (ID: NG_047232.1), Mucilaginibacter sp. PAMC 26640 (ID: CP014772.1), Campylobacter concisus pCCON16 (ID: NC_009796.1), Ralstonia pickettii 12D pRp12D02 (ID: NC_012849.1), Enterobacter cloacae pEC005 Class A EBSL TEM-157 (ID: NG_050201.1), Cupriavidus metallidurans CH34 megaplasmid (ID: NC_007974.2), Streptococcus pneumoniae pSpnP1 (ID: NC_008350.1) and Acinetobacter baumannii str. AYE plasmid p1ABAYE (ID: NC_010401.1). A plus sign “ + ” denotes the presence of the plasmid in the sample.

### Detection of plasmid residues in commercially available polymerases

To evaluate whether plasmid residues are commonly present in commercially available polymerase preparations, we tested *Taq* polymerases (n = 4), high-fidelity polymerases (n = 2) and qPCR mastermixes (n = 7) for the presence of an origin of replication (pBM1/pUC19/pBR322/ColE1) and selection markers (*bla*_*TEM-1*_*; CmR*). An origin of replication and an ampicillin resistance had been found in two polymerase preparations (HotStarTaq, EvaGreen). An origin of replication had only been found in one polymerase preparation (iTaq Universal Probes Supermix). A Chloramphenicol resistance had not been found in any of the polymerase preparations tested. The methodology used did not incorporate a negative control to see if a positive signal can be obtained. Therefore, possible laboratory cross-contamination could not be excluded entirely although being unlikely due to PCR mastermix preparation in CleneCab PCR Workstation and highly specific primers. (Herolab, Wiesloch, Germany). To confirm our findings, enzymes preparations that had been tested positive for plasmid residues were used as template and amplified with a previously plasmid negative polymerase preparation, (GoTaq G2 Hot Start Polymerase; Promega). The HotStarTaq was still positive for Ori- and Ampicillin presence and the EvaGreen 2X qPCR Express Mix-ROX remained only positive for Ori presence, indicative for possible presence of artificial expression plasmids. All previous positive tested *Taq* enzymes from BioRad had been tested negative and, therefore, reconfirmed negative for plasmid presence (Table 2).

**Table 2:** Testing of commercial available enzymes for presence of plasmid sequences. The first test (PCR reaction w/o template) was run without any template. The mastermix contained all necessary additives as well as specific primers. A minus sign “−” denotes a negative result, whereas a plus sign “+” shows a positive result. Enzymes tested negative in the first experiment were not tested in the second experiment and results are therefore not available (N/A). If an enzyme was tested positive in the first run, 0.25 µl of the polymerase was added as template to a mastermix tested previously negative. If the results were positive the enzyme added as template contained plasmid residues.

Polymerases	Plasmid Targets					
	Ori		Bla_Tem-1_		CmR	
	PCR reaction w/o Template	Enzyme Preparation as Template	PCR reaction w/o Template	Enzyme Preparation as Template	PCR reaction w/o Template	Enzyme Preparation as Template
^a^HotStarTaq DNA Polymerase	+	+	+	+	−	N/A
^b^iTaq DNA Polymerase	−	−	−	−	−	N/A
^c^Taq DNA Polymerase	−	N/A	−	N/A	−	N/A
^d^GoTaq G2 Hot Start Polymerase	−	N/A	−	N/A	−	N/A
^c^Phusion High-Fidelity DNA Polymerase	−	N/A	−	N/A	−	N/A
^b^iProof High-Fidelity DNA Polymerase	−	N/A	−	N/A	−	N/A
^c^Luna Universal qPCR Master Mix	−	N/A	−	N/A	−	N/A
^c^Luna Universal Probe qPCR Master Mix	−	N/A	−	N/A	−	N/A
^c^Luna Universal Probe One-Step RT-qPCR Kit	−	N/A	−	N/A	−	N/A
^d^GoTaq Probe qPCR Master Mix	−	N/A	−	N/A	−	N/A
^e^EvaGreen 2X qPCR Express Mix-ROX®	+	+	+	−	−	N/A
^b^iTaq Universal Probes Supermix	+	−	+	−	−	N/A
^f^TaqMan Universal PCR Master Mix	−	N/A	−	N/A	−	N/A

^a^Enzymes sold by Qiagen; ^b^Enyzmes sold by BioRad; ^c^Enzymes sold by New England Biotechnologies; ^d^Enyzmes sold by Promega, ^e^Enzymes sold by ABM Life Sciences; ^f^Enzymes sold by Thermofisher.

#### Analysis of metagenomics studies

Finally, we analyzed previously published metagenomic data sets of human gut and plasma samples as well as a data set using different whole genome amplification kits^50–52^ for the presence of plasmid residues. Retrospective analysis of these data sets, natural plasmid residues had been found in most sets and most commonly *Acinetobacter sp*. and *Escherichia sp*. as source organisms (Table 1 and Table 2). The highest diversity of plasmids had been found in metagenomic data focusing on the fecal microbiome^53^. Especially metagenomic studies analyzing high bio mass samples such as microbiome studies are expected to contain a higher amount and diversity of natural plasmids compared to samples with low biomass (e.g. plasma). Remarkably, a plasmid highly similar to *Xuhuaishuia manganoxidans* strain DY6-4 had been detected in several samples of two unrelated metagenomics studies although this bacterium has been found only in the Pacific Clarion-Clipperton Fracture Zone^51^ (Table 3) so far.

**Table 3:** Analysis of published metagenomic data for foreign plasmids. In all analyzed metagenomics studies albeit not in all of the samples, plasmid sequences were found. The same plasmid sequences found in the metagenomic data set of J.R. Bedarf *et al*. and M. Thoendel *et al*., were *Bacteroides fragilis* IB143 (ENA ID: U30316.1), tetracyclin-coding resistance of *Bifidobacterium longum* B200304 pBIF10 (ENA ID: DQ093580.1) and *Xuhuaishuia manganoxidans* strain DY6-4 plasmid (ENA ID: CP019124.1). Unique plasmid sequences found for J. R. Bedarf *et al*., were *Streptococcus parasanguinis* plasmid pFW213 (Accession: NC_012642.1), *Escherichia coli* plasmid pCM959 (ENA ID: K00826.1) *Bacteroides thetaiotaomicron* VPI-5482 plasmid p5482 (ENA ID: GCA_000011065.1), *Escherichia coli* strain D1 plasmid A (ENA ID: CP010134.1), *Escherichia coli* strain MDR_56 plasmid unnamed 5 (ENA ID: CP019906.1), *Eubacterium eligens* ATCC 27750 plasmid (ENA ID: 515620) and *Pseudomonas chlororaphis* strain PCL1606 plasmid (ENA ID: CP011110.1) as well as *Staphylococcus epidermidis* plasmid SAP108D (ENA ID: GQ900461.2) for M. Thoendel *et al*. In the sequences of J. Law *et al*., although using unspecific pre-amplification with different MDA Kits only *Acinetobacter pittii* strain AP_882 plasmid pNDM-AP_882 (ENA ID: CP014477.1), *Acinetobacter soli* strain GFJ2 plasmid pGFJ6 and pGFJ7 (ENA: CP016902.1) were found.

Plasmid ID	Publication		
	J Bedarf & F Hildebrand *et al*.	J. Law *et al*.	M. Thoendel *et al*.
*Bacteroides fragilis* IB143	+		+
*Streptococcus. parasanguinis* pFW213	+		
*Escherichia coli* pCM959	+		
*Bifidocbacterium longum* pBIF10 tet(Q) gene	+		+
*Bacteroides thetaiotaomicron* p5482	+		
*Escherichia coli* D1 plasmid A	+		
*Escherichia coli* MDR 56 plasmid unnamed	+		
*Eucbacterium eligens* ATCC 27750	+		
*Xuhuaishuia manganoxidans* strain DY6-4	+		+
*Pseudomonas chlororaphis* strain PCL1606			+
*Staphylococcus epidermidis* SAP108D			+
*Acinetobacter pittii strain* AP_882 pNDM-AP_882		+	
*Acinetobacter soli strain* GFJ2 plasmid pGFJ6		+	
*Acinetobacter soli strain* GFJ2 plasmid pGFJ7		+	

## Discussion

The presence of bacterial DNA residues in commercially available enzymes, DNA extraction kits and other molecular grade reagents have been recognized recently^21,26,41,52^. The presence of plasmids in molecular biology reagents, however, has remained unnoticed, so far. We found natural and artificial plasmid residues in most tested NGS reagents including particularly recombinant generated enzyme preparations. Sources of these plasmids included laboratory contaminants as well as bacteria and expression vectors used for the generation of recombinant proteins. Plasmid sequences have been identified frequently in NGS studies, but may have been attributed erroneously to bacteria. Hence, plasmid sequences present in clinical and environmental samples may have far-reaching consequences.

Metagenomic studies are increasingly used in addition to standard PCR assays to address clinical questions as reviewed in Klymiuk & Steininger^54^. Enzymes used for these assays are generated by recombination in (with) prokaryotic systems. Plasmid sequences may misguide clinical treatment decisions and adversely affect patient outcome. For example, antimicrobial resistance testing is increasingly adjunct by testing bacterial isolates for the presence of genes that confer resistance^55^. In the studies analyzed, common antibiotic resistance gene sequences had been found from *Enterobacter cloacae* and *Serratia marcesens*. These two pathogens are increasingly resistant to multiple or most antimicrobial drug classes and the presence of resistance genes in clinical samples would not be surprising or questioned^14,15,17,45^. Consequently, the choice of antimicrobial treatment would be misguided towards reserve antimicrobials that are more toxic than standard ones. At least one patient death was documented in association with a false-positive test result by a contaminated mastermix^56^.

Misguidance of clinical decisions may also be associated with false-positive PCR results. We found evaluated EIAV sequences in all human samples. We could identify the plasmid used for the generation of the reverse transcriptase as the source of these sequences. Identification of a horse retrovirus in human samples was implausible, which guided our investigation into the right direction. In general, the presence of host-specific viral, genomic or plasmid DNA (e.g. *Xuhuaishuia manganoxidans* strain DY6-4) in samples derived from other hosts should be questioned for their plausibility. Still, recombinant reverse transcriptase is also used in PCR assays for detection of EIAV in horse samples and this *pol* sequence is used in several detection assays as target^57^. A positive test result would be plausible and negative controls would test negative because they are usually not treated with a reverse transcriptase. In case of a single positive EIAV test result, however, all horses of the stable would be culled.

Elimination of plasmid sequences from molecular biology reagents is difficult and costly. The presence of natural plasmids from bacteria such as *Ralstonia sp., Bradyrhizobium sp*. and *Legionalla sp*., are common contaminants in Ultrapure Water and are difficult to avoid^21^. Contamination of reagents from the human body may remain unnoticed. In one of our recent metagenomic studies, we found plasmid fragments from *Ralstonia sp*., *Burkholderia sp*., *Enterobacter sp*., *Acinetobacter sp*., and *Micrococcus sp*.^48^. The first two were likely introduced by water samples, whereas the later were likely introduced through human handling as these microbes are part of the normal human skin flora^58^. Previously, we found Bicaudal-1 and UL32 protein expression plasmids in human samples^48^. These plasmids were very likely contaminations as our research group used these plasmids in another research study. In addition, prokaryotic expression plasmids are commonly used to generate enzymes for molecular biology and are difficult to eliminate. For example, we identified the plasmid used for the generation of the EIAV reverse transcription as the pDS56/RBSII-based plasmid expression vector by the backbone^59^. Nevertheless, we also found differences in the level of contamination between the enzyme preparations from different manufacturers, which also indicates the feasibility of reducing this background signal.

A possible, inexpensive and feasible solution to the problem of plasmid residues in metagenomics studies may be the testing of technical replicates of the samples as well as the negative controls in parallel and subtracting during bioinformatics analysis signals detectable in both samples. Databases that comprehensively annotate the different expression vectors used for recombinant generation of proteins are important in this respect. Furthermore, specification of the type and sequences of expression plasmids used in the package inserts of every molecular biology reagent would be helpful. Nevertheless, most production processes of enzymes are proprietary and, in our experience, companies are very hesitant to provide this information.

Another solution, presented by de Goffau and colleagues, would be to use different isolation kits during sample preparation to control if the results are reproducible^60^.

In conclusion, we found that plasmid sequences are frequently present in molecular biology reagents. The sources for this background noise in metagenomic studies are diverse and include contamination of reagents from the environment, cross-contamination in the laboratory from purposely generated plasmids, as well as plasmids used for the generation of enzymes. The amount and type of plasmids found in metagenomics studies may greatly vary upon pre-treatment of samples (e.g. use of different enzymes). The presence of these plasmids in samples may have far-reaching consequences including the misguidance of therapeutic decisions in human and veterinary medicine – particularly when unexpected. Our observations open up whole new avenues to identifying and appropriately addressing these potential issues. Background plasmid noise may be eliminated for example from signals by use of appropriate negative controls, manufacturers of enzymes and recombinant proteins may inform customers of the possible presence of plasmid traces, and metagenomic data will be interpreted even more cautiously.

## Methods

Urine and pharyngeal lavage samples from human healthy volunteers had been collected in a sterile collection cup (Greiner Bio-One GmbH, Kremsmünster, Austria) as described previously^48^. Lavages had been collected by asking the patient to gurgle with 10 ml of sterile, physiologic sodium-chloride solution (0.9% NaCl Mini-Plasco isotonic solution, B. Braun-Austria GmbH, Maria Enzersdorf, Austria) for a minimum of one minute and collecting the lavage fluid in a sterile tube. Samples had been kept on ice and had directly been processed. Nucleic acids had been enriched with Vivaspin 20 50.000 MWCO PES ultracentrifugation columns (Sartorius, Aubagne, France) at 4000 g and 4 °C. Total DNA and RNA had then been purified with the Roche High Pure Viral Nucleic Acid Kit (Roche, Mannheim, Germany) and reverse transcribed with either iScript cDNA Synthesis Kit (Bio-Rad, Hercules, USA) or Omniscript RT Kit (Qiagen, Hildesheim, Germany) according to the manufactures instructions. The samples had been cryopreserved at −80 °C until testing.

### Plasmid detection

For the detection of Equine Infectious Anemia Virus *pol* sequences, a PCR assay had been developed amplifying a 723 bp long fragment of the *pol* gene of EIAV and corresponds to the fragment detected previously with use of the following primers: forward: 5′-CGG-AAG-AGG-CAC-AAA-AAG-AG-3′; reverse: 5′-GAC-CAG-GTA-CCC-AAG-CAA-AA-3′. The PCR mix contained 0.125 µl OneTaq Hot Start DNA Polymerase (New England Biosystems, Ipswich, USA), 500 µM of each primer, 5 µl ThermoPol Buffer (New England Biosystems; Ipswich, USA), 200 µM of each dNTP (ThermoFisher, Waltham, USA) and 1 µl DNA or cDNA template per 25 µl reaction mix. Amplification started with an initial denaturation step at 94 °C for 5 minutes, followed by 40 cycles of denaturation at 94 °C for 60 seconds, annealing at 51 °C for 30 seconds and extension at 68 °C for 60 seconds, followed by a final extension time of 7 minutes The PCR product had then been visualized on a 1.0% agarose gel in Tris-Acetat-EDTA (TAE) with a ChemiDoc XRS + System (Biorad, California, USA).

For quantitative analyses of plasmid copies, a qPCR assay amplifying in part the chloramphenicol acetyltransferase (CmR) encoding gene had been designed with the use of the online-tool GenScript Real-time PCR (TaqMan) Primer Design (https://www.genscript.com/ssl-bin/app/primer). The 20 µl reaction mix contained 9 µl iTaq Universal Probes Supermix (Bio-Rad, Hercules, USA), 300 nM primers (Forward: 5′-GAC-GGT-GAG-CTG-GTG-ATA-TG-3′; Reverse: 5′-TGT-GTA-GAA-ACT-GCC-GGA-AA-3′), 200 nM of the CmR Probe (5′-FAM-CGC-TCT-GGA-GTG-AAT-ACC-ACG-ACG-TAMRA-3′) and 5 µl template. The reaction had been done in a 96-well optical microtiter plate (Life Technologies, Carlsbad, CA, USA) and amplified in a StepOnePlus Real-Time PCR System (Thermo Fisher Scientific, Waltham, MA, USA). The reaction mix had been pipetted into a MicroAmp Fast 96-Well Reaction Plate 0.1 ml (Applied Biosystems, California, USA) and afterwards 5 µl of template had been added. The cycling conditions included an initial denaturation step at 95 °C for 2 minutes, followed by 40 cycles of denaturation for 15 seconds at 95 °C and 20 seconds extension time at 60 °C. Every run of the CmR qPCR included a serial dilution of the plasmid pDONR221 from 3 × 10^1^ to 3 × 10^6^ copies per well for calculation of a standard curve and quantification of target sequences. Each DNA sample had been analyzed in triplicate and at least 12 negative controls, only containing the reaction mix with 1 µl ddH_2_0 as template, had been included in each run.

In order to test commercially available polymerases for presence of plasmid sequences, a specific pan-Ori primer pair (Forward: 5′-AGT-TCG-GTG-TAG-GTC-GTT-CG-3′; Reverse: 5′-GCC-TAC-ATA-CCT-CGC-TCT-GC -3′) had been designed with the online primer design tool Primer3 v.0.4.0. (http://bioinfo.ut.ee/primer3-0.4.0/primer3/). This PCR assay allowed detection of pBM1, pBR322, ColE1 and pUC19 in one reaction. The commonly used penicillin resistance bla_TEM-1_, had been detected by a PCR using a primer pair designed by Lee and colleagues (Forward: 5′-CTA-CGA-TAC-GGG-AGG-GCT-TA-3′, Reverse: 5′-ATA-AAT-CTG-GAG-CCG-GTG-AG-3′)^53^. For the detection of Chloramphenicol resistance (CmR) the same primer pair had been used as for the described qPCR. Cycling conditions and set up of reaction mixes had been conducted according to the enclosed manufacturer’s manual except that no template had been added. All PCR reactions consisted of 30 cycles with 30 seconds denaturation at 95 °C, 30 seconds annealing at 60 °C and 25 seconds extension time at 72 °C. The time needed for initial denaturation and final extension as well as primer, MgCl_2_ and dNTP concentration may vary upon polymerase or mastermix used. Cycling conditions for High-Fidelity Polymerases such as Q5 and iProof were shorter (10 seconds denaturation and 20 seconds extension time). As positive control for Ampicillin and Ori presence, 1 µl of a 1 ng/µl pcDNA3.1(+) dilution has been used as template. The (RT)-qPCR mastermixes had been pipetted according to each manufacturer’s manual. The same cycling conditions had been used as for the PCR reaction.

To exclude false-positive results, 0.125 µl to 0.2 µl of pure enzyme had been used as template for amplification with the GoTaq G2 DNA Polymerase (Promega, Madison, Wisoconsin, USA) which had no detectable plasmid residues. Cycling conditions included a 2 minute initial denaturation step at 95 °C, followed by 30 cycles of 30 seconds denaturation at 95 °C, 30 seconds annealing at 60 °C and 25 seconds extension time at 72 °C with a final extension time of 5 minutes at 72 °C. A 25 µl reaction consisted of 5 µl Colorless Flexi Buffer, 0.5 µl of each 10 µM primers, 0.5 µl of 10 µM dNTP Mix, 0.125 µl GoTaq G2 Enzyme and 2.5 µl 25 mM MgCl_2_^61^. Positive tested qPCR Mastermixes had been reevaluated, by testing a 50 µl reaction instead of 25 µl in order to incorporate more enzymes.

Every mastermix had been pipetted in a CleneCab PCR Workstation (Herolab, Wiesloch, Germany) to avoid the introduction of foreign nucleic acids. The templates had been added on ice while the clean cab itself had been decontaminated for 20 min with the use of UV irradiation.

### Bioinformatics and Statistical Evaluation

The EIAV *pol* sequence detected in our previous study has been analyzed in comparison with reference sequences including EIAV *pol* Wyoming (ID: AF016316.1), EIAV *pol* Liaoning (ID: AF327877.1), EIAV *pol* Vaccine Strain (ID: gb|AF327878.1), EIAV *pol* V70 Strain (ID: gi 9929860), EIAV *pol* V26 Strain (ID: gi 9929867), EIAV *pol* (ID: NC_001450.1), EIAV *pol* Clone 22 (ID: M87581.1), EIAV *pol* Miyazaki2011-A (ID: JX003263.1) and other strains of the *lentiviridae* group such as Human Immunodeficiency Virus-1 *pol* (HIV-1; ID: NC_001802.1), Feline Immunodeficiency Virus *pol* (FIV; ID: NC_001482.1) and Maedi/Visna *pol* strain kv1772 (ID: NC_001452.1) with use of the software package CLC Main Workbench 7 (Qiagen, Hildesheim, Germany).

In order to evaluate contigs for further potential plasmid contaminations, sequences had been evaluated for the presence of common plasmid features including origin of replication (F1, pBR322, pUC19, p15a, ColE1, SV40), selection markers (Chloramphenicol, Ampicillin (Bla_Tem-1_), Kanamycin (*Tn5*), Streptomycin (*aadA*), Puromycin (*pac*) and Hygromycin (*hph*)), promoter (T7, T3, Sp6, AmpR, CMV, tet, LacI, polyhedrin, SV40), terminator (rrnB T1-T2, lambda), protein tags (Histidine, HA, Streptavidin) and primer binding sites (pBluescript SK, pBluescript KS, M13 pUC and other commonly used primer sites). All plasmid sequences had been searched from 5′ to 3′as well as from 3′to 5′. Sequences with at least one of these characteristics had been analyzed further by the SnapGene Viewer software (GSL Biotech LLC, Chicago, USA), which automatically annotates plasmid features. All sequences attributed to plasmids had been analyzed via their annotated features and classified into artificial vectors or artificial plasmid fragments (see Fig. 5A).

As final step, known plasmid sequences had been searched in the short read metagenome sequence data of all samples, which was described earlier by Thannesberger and colleagues^48^ as well as published raw data from other metagenomics studies^50–52^. We used the previously described bioinformatic pipeline^48^ which estimates the coverage along the plasmids and rejects short regions of unspecific coverage. All plasmid sequences from the NCBI RefSeq database, release 77, had been used as reference^54^.

### Ethics Statement

All healthy donors of clinical samples (urine and saliva) provided written informed consent. All experimental protocols had been approved by the Medical University of Vienna.

### Method Statement

All methods had been carried out in accordance with relevant guidelines and regulations.

### Abbreviated Summary

Due to increasing sequencing throughput enabled through Next-Generation sequencing (NGS), the analysis of all microbial genomes present in a single sample became possible (metanogemics). The indiscriminant sequencing of all nucleic acid sequences present in a sample by metagenomics does pose the risk of attributing biological significance to contaminating sequences as well as biasing the biological signal through a technical signal. Thus research conclusions and clinical decisions may be misguided significantly. We found that background plasmid sequences are present in every biological sample and have been erroneously interpreted as clinically significant biological differences previously. Through recognition of this significant background in metagenomic studies, however, we show how to devise effective countermeasures such as labelling of commercial reagents for presence of plasmids used for generation of recombinant proteins, and specifying these.

## Supplementary information

Supplementary information
