# Supplementary material for: Plasmid DNA contaminant in molecular reagents
Source: Sci Rep. 2019 Feb 7;9:1652. doi: 10.1038/s41598-019-38733-1 (PMC6367390; doi:10.1038/s41598-019-38733-1)

Plasmid DNA contaminant in molecular reagents

Running head: Plasmid Residues in Enzyme Preparations

Wally N[1] , Schneider M[1] , Thannesberger J[1] , MT Kastner[1] , Bakonyi T[2] , Indik S[2] , Rattei T[3] , J Bedarf[4] , F Hildebrand[5], J. Law[6] , J Jovel[6], Steininger C[1*]

Affiliations:

[1]Division of Infectious Diseases, Department of Medicine 1, Medical University of Vienna, Vienna, Austria

[2]University of Veterinary Medicine, Department of Virology, Vienna, Austria

[3]CUBE-Division of Computational Systems Biology, Department of Microbiology and Ecosystem Science, University of Vienna, Vienna, Austria

[4]German Centre for neurodegenerative disease research (DZNE), Department of Neurology, University of Bonn, Bonn, Germany

[5]European Molecular Biology Laboratory, EMBL, Heidelberg, Germany

[6]Department of Medicine, University of Alberta, Edmonton, Alberta, Canada

***Corresponding author:**

Christoph Steininger, MD; Medical University Vienna; Department of Medicine I

Währinger Gürtel 18-20; 1090 Vienna – Austria; T: +43 – 1- 40 400 44400; F: +43 – 1- 40 400 44180; E-mail: christoph.steininger@meduniwien.ac.at (CS)

Funding:

This study was supported by a research grant from the Austrian Science Fund P25353-B21 and P28102-B30.

Keywords: Background noise, Next-generation sequencing, Diagnostic assays, Metagenomics, Plasmids, Specificity

Author List

1. Nikolai Wally (First Author)

Division of Infectious Diseases, Department of Medicine 1, Medical University of Vienna, Vienna, Austria

E-Mail: n.wally@gmx.at

Telephone Nummber: +43 660 1455121

1. Martina Schneider

Division of Infectious Diseases, Department of Medicine 1, Medical University of Vienna, Vienna, Austria

E-Mail: martina.a.schneider@meduniwien.ac.at

2. Jakob Thannesberger

Division of Infectious Diseases, Department of Medicine 1, Medical University of Vienna, Vienna, Austria

E-Mail: jakob.thannesberger@meduniwien.ac.at

3. Marie-Theres Kastner

Division of Infectious Diseases, Department of Medicine 1, Medical University of Vienna, Vienna, Austria

E-Mail: marie-theres.kastner@meduniwien.ac.at

4. Tamas Bakonyi

University of Veterinary Medicine, Department of Virology, Vienna, Austria of Veterinary Medicine, Department of Virology, Vienna, Austria

E-Mail: Tamas.Bakonyi@vetmeduni.ac.at

5. Stanislav Indik

University of Veterinary Medicine, Department of Virology, Vienna, Austria of Veterinary Medicine, Department of Virology, Vienna, Austria

E-Mail: Stanislav.Indik@vetmeduni.ac.at

6. Thomas Rattei

CUBE-Division of Computational Systems Biology, Department of Microbiology and Ecosystem Science, University of Vienna, Vienna, Austria

E-Mail: thomas.rattei@univie.ac.at

Janis Bedarf

German Centre for neurodegenerative disease research (DZNE), Department of Neurology, University of Bonn, Bonn, Germany

E-Mail: Janis.Bedarf@ukbonn.de

Falk Hildebrand

European Molecular Biology Laboratory, EMBL, Heidelberg, Germany

E-Mail: falk.hildebrand@googlemail.com

Division of Infectious Diseases, Department of Medicine 1, Medical University of Vienna, Vienna, Austria

Janis Jovel

Department of Medicine, University of Alberta, Edmonton, Alberta, Canada

E-Mail: jovel@ualberta.ca

John Law

Department of Medicine, University of Alberta, Edmonton, Alberta, Canada

E-Mail: llaw@ualberta.ca

Christoph Steininger

Division of Infectious Diseases, Department of Medicine 1, Medical University of Vienna, Vienna, Austria

E-Mail: christoph.steininger@meduniwien.ac.at

Telephone Number: +43 660 121 70 17

Raw Data

Fig. 3A

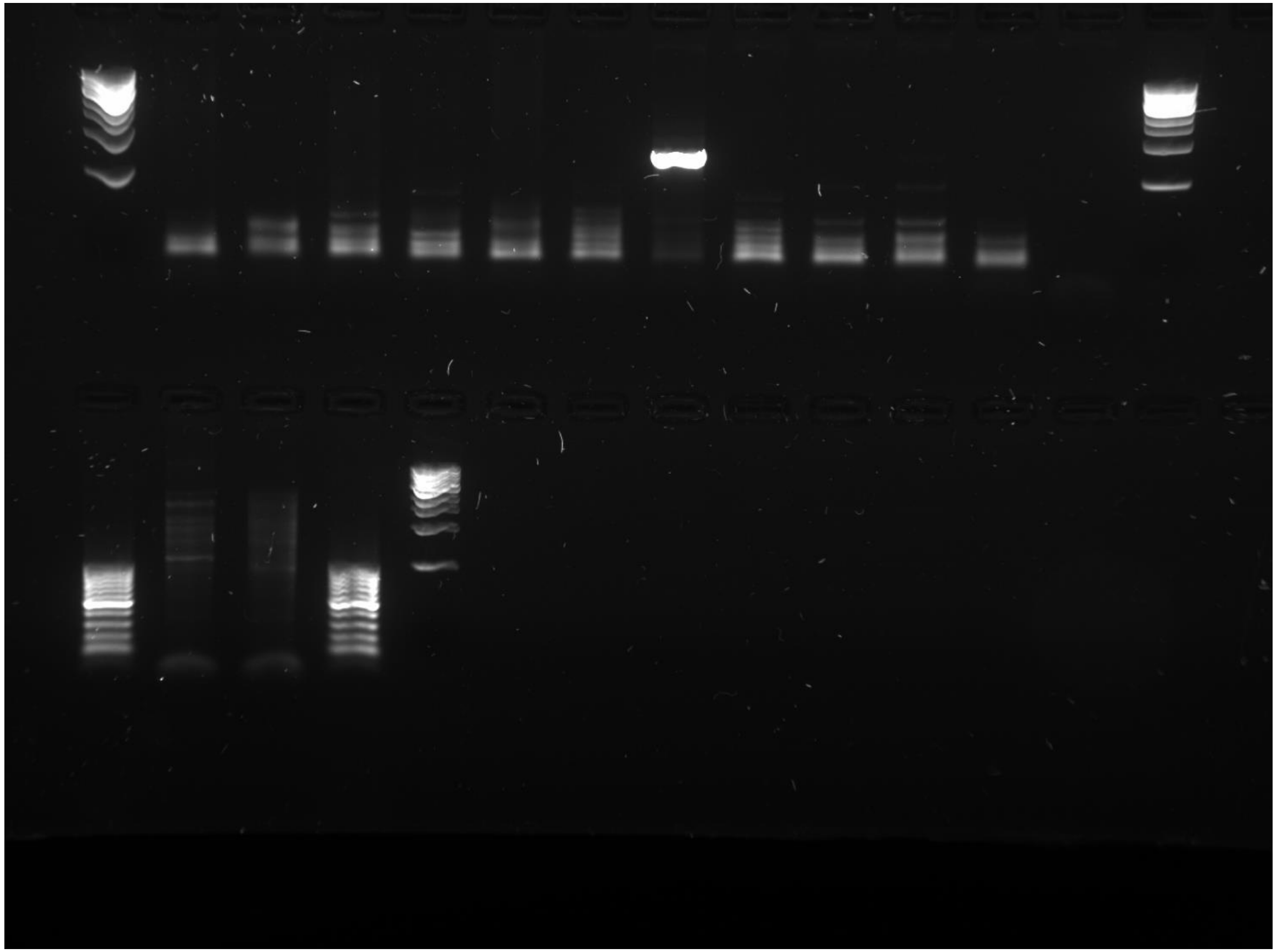

Fig. 3B

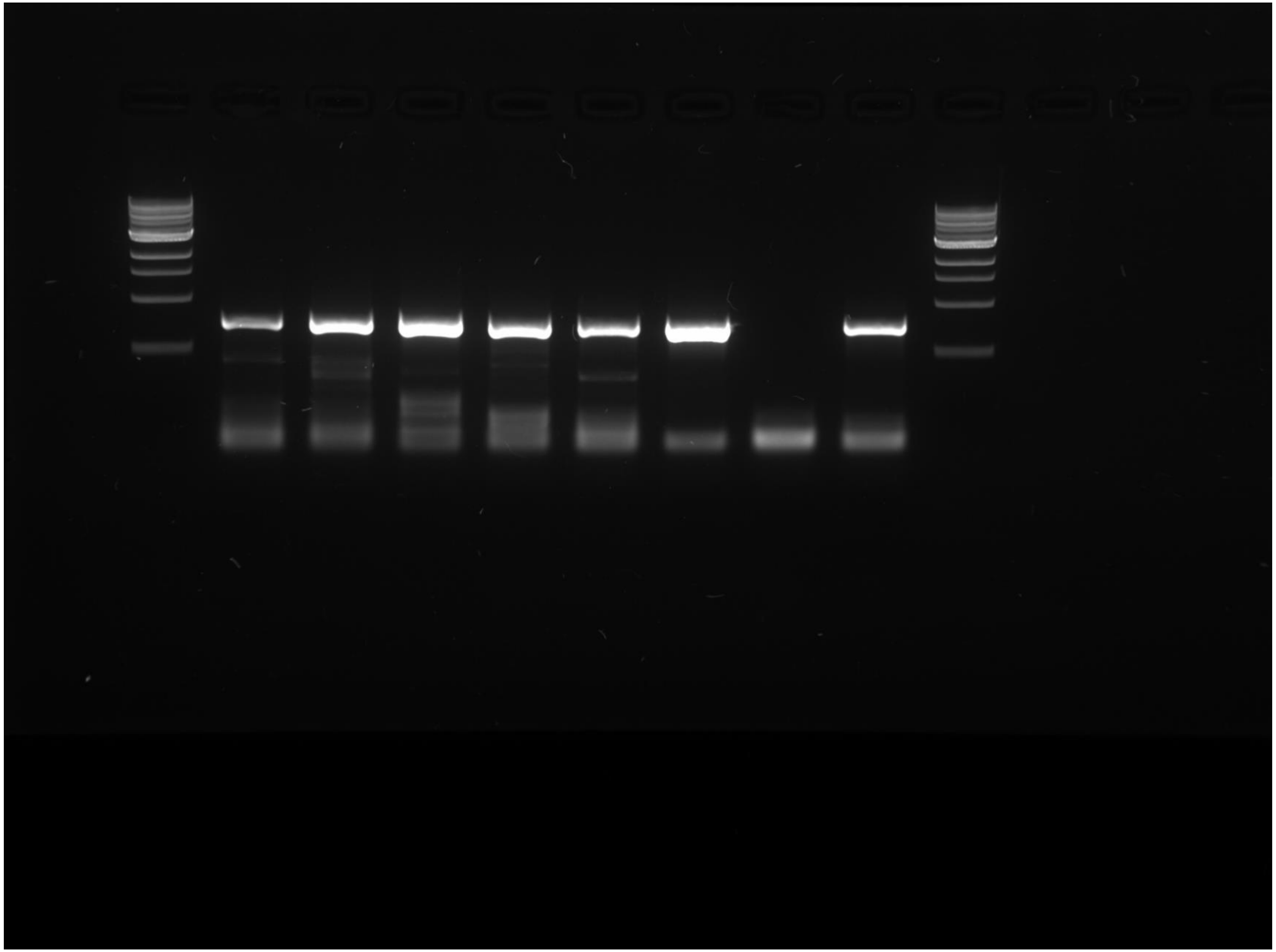

Supplement: Supplementary file 1 — Supplementary information [file 41598_2019_38733_MOESM1_ESM.pdf]
